# Left Ventricular Function and Echocardiographic Parameters in Patients Undergoing Chemotherapy With 5‐Fluorouracil, Anthracycline, Taxotere, and Herceptin: Prospective Cohort Study

**DOI:** 10.1002/hsr2.71031

**Published:** 2025-07-27

**Authors:** Alireza Abdollahi Moghadam, Seyed Hamed Fazaeli, Mostafa Kamandi

**Affiliations:** ^1^ Department of Cardiovascular Disease, Faculty of Medicine Mashhad University of Medical Sciences Mashhad Iran; ^2^ Department of Internal Medicine, Faculty of Medicine Mashhad University of Medical Sciences Mashhad Iran

**Keywords:** anthracycline, cancer, cardiotoxicity, chemotherapy, herceptin, left ventricular function

## Abstract

**Background:**

Despite recent progress in cancer treatment, the side effects of treatment are recognized as a major source of mortality and morbidity in patients. This study was developed and conducted to evaluate the influence of chemotherapy on left ventricular function and echocardiographic and electrocardiographic parameters in patients undergoing chemotherapy with 5‐fluorouracil, anthracycline, taxotere, and herceptin.

**Methods:**

This study is a prospective cohort study on patients diagnosed with cancer. After reviewing the inclusion and exclusion criteria, patients referred for treatment with 5‐fluorouracil, anthracycline, taxotere, or herceptin were randomly divided into study groups. Echocardiographic and electrocardiographic parameters such as left ventricular ejection fraction (LVEF), LV end systolic dimension (LVESD), LV end diastolic dimension (LVEDD), tricuspid annular plane systolic excursion (TAPSE), Mid RV diameter, RA volume, QRS duration, and QTc were evaluated at the beginning of the study and 6 months after starting treatment. Subsequently, the measurements were compared and analyzed.

**Results:**

Overall, 80 patients undergoing chemotherapy with 5‐fluorouracil, anthracycline, taxotere, or herceptin were included in this study. LVEF and TAPSE were significantly lower after 6 months in patients treated with anthracycline and herceptin, while LV end diastolic dimension and LV end systolic dimension were higher significantly (*p*‐value < 0.05). In patients treated with 5‐fluorouracil and taxotere, a significant difference in TAPSE was seen after 6 months, but no significant difference was found in LVEF or other echocardiographic parameters (*p*‐value > 0.05).

**Conclusion:**

The findings indicated that chemotherapy with anthracycline and herceptin is significantly linked to altered left ventricular function and echocardiographic measures such as LV end diastolic dimension, LV end systolic dimension, and TAPSE. However, future research with more extensive follow‐up are recommended to investigate cardiac side effects in these patients.

## 1 Introduction

Recent advances in cancer detection and more effective treatments have led to increased survival of cancer patients [1]. Meanwhile, the incidence of cancer is expected to increase by up to 70% over the next 20 years, according to the World Health Organization [2]. Cardiovascular diseases and cancer are the two main causes of morbidity and mortality worldwide, accounting for at least 70% of medical causes of death globally [3]. Along with the increasing number of patients experiencing long‐term survival after a cancer diagnosis, the number of patients suffering from adverse side effects of anticancer drugs is also rising [3, 4].

Echocardiography serves as a primary modality for the surveillance of cardiac function during and following oncological treatments, particularly for assessing left ventricular systolic performance and identifying cardiotoxicity [5]. Diastolic dysfunction has emerged as a critical prognostic indicator for overall mortality and is a significant contributor to the onset of heart failure (HF). Some investigations have reported the appearance of diastolic abnormalities preceding the development of systolic impairment in individuals undergoing treatment for breast cancer [6, 7]. In contrast, alternative studies examining the cardiac consequences of chemotherapy in breast cancer patients have not demonstrated a measurable effect on diastolic function [8]. Although diagnostic thresholds for chemotherapy‐induced systolic dysfunction have been defined, standardized guidelines and criteria for identifying diastolic dysfunction caused by medical intervention are still lacking.

Emerging evidence points to the enhanced sensitivity of global longitudinal strain (GLS) over traditional left ventricular ejection fraction (LVEF) in detecting early systolic impairment, predicting latent cardiotoxic effects, and guiding heart failure management during anthracycline therapy [7, 9, 10, 11, 12]. Within the context of evaluating left ventricular diastolic function, parameters such as GLS, the E/e' ratio, and the left atrial volume index (LAVi) have demonstrated considerable diagnostic utility. However, distinguishing normal from impaired diastolic function via noninvasive techniques remains a considerable clinical challenge.

In patients who retain normal systolic function (LVEF > 50%), current recommendations highlight four diagnostic indicators: septal e' velocity below 7 cm/s or lateral e' below 10 cm/s, an E/e' ratio exceeding 14, LAVi greater than 34 mL/m², and a peak tricuspid regurgitation velocity over 2.8 m/s. A diagnosis of diastolic dysfunction is made when more than half of these metrics exceed the specified thresholds [13]. Nonetheless, many primary studies and systematic reviews addressing diastolic evaluation during and after chemotherapy and radiotherapy tend to omit a thorough grading of diastolic impairment [8, 9, 14, 15]. One promising approach for the early recognition of subclinical diastolic dysfunction involves analyzing the propagation velocity of intrinsic transmitral flow, a parameter that reliably reflects the dynamics of ventricular filling [16].

A wide range of cancer therapies (e.g., cyclophosphamide, tyrosine kinase inhibitors (TKIs), trastuzumab, anthracyclines, 5‐fluorouracil, proteasome inhibitors, taxotere, and immune checkpoint inhibitors) have been linked to a higher likelihood of both acute and delayed cardiovascular toxicity and related diseases [17, 18, 19]. These adverse cardiovascular outcomes encompass a broad spectrum of conditions, such as hypertension, left ventricular dysfunction, acute coronary syndromes, myocardial infarction, atrial and ventricular arrhythmias, prolonged QT intervals, pericardial effusion, and HF, as well as pulmonary complications [17, 20, 21]. Congestive heart failure due to chronic cardiac toxicity is the most common type of injury caused by these drug regimens, especially anthracycline agents, causing irreversible damage that peaks 1–3 months after drug administration [22]. Myocardial injury often occurs as a result of increased levels of oxygen free radicals and lipid peroxidation in the myocardium, which contribute to myocardial injury [6, 19]. Therefore, early diagnosis of patients who develop left ventricular dysfunction following anticancer drug therapy is of high clinical importance [23]. The incidence and severity of cardiotoxic effects are influenced by the specific chemotherapy regimen, comorbid conditions, duration, and patient risk profiles. These may interact to amplify the overall cardiotoxic burden. The current findings support the utility of N‐terminal pro‐B‐type natriuretic peptide (NT‐proBNP) as a valuable biomarker in detecting early cardiac injury; however, extended follow‐up periods are necessary to detect the trajectory of LVEF changes and the onset of HF. In light of these observations, initiating therapy with a comprehensive biomarker panel, including high‐sensitivity troponin and NT‐proBNP is advisable [24, 25, 26].

Detecting cardiotoxicity solely through reductions in LVEF may be problematic due to its delayed manifestation and the limited efficacy of therapeutic interventions at that stage [27, 28, 29]. Patients with pre‐existing cardiovascular conditions are particularly vulnerable to these complications [3]. Given the scarcity of robust data on the interplay between chemotherapeutic protocols and ventricular function, this investigation was undertaken to evaluate the impact of different chemotherapy regimens on left ventricular performance, along with associated echocardiographic and electrocardiographic findings.

## 2 Materials and Methods

This study utilized a prospective cohort design. The conduct of the study complies with the Declaration of Helsinki and received approval by the Institutional Ethics Committee of Mashhad University of Medical Sciences (code: IR.MUMS.MEDICAL.REC.1401.022). Participants were randomly selected from cancer patients referred to the cardiology department and cardiology clinic of Imam Reza Hospital (Mashhad, Iran) during 2021‐2022 for treatment with one of the following chemotherapy drugs: 5‐fluorouracil, anthracycline, taxotere, or herceptin.

The inclusion criteria were: age > 18 years, no history of heart disease, normal LVEF (> 55%), absence of wall motion abnormalities and left ventricular dilatation on initial echocardiography, absence of cardiac conduction blocks (AV blocks and bundle branch blocks), normal QTc, absence of ST‐T segment changes, and normal cardiac axis. The exclusion criteria were: lack of consent and failure to meet the inclusion criteria.

Initial patient information, including demographic and clinical variables and risk factors for cardiovascular disease, was collected through a checklist at the beginning of the study. Echocardiography was performed by a cardiologist using a VIVID S6 device with a 2.5 MHz probe. Echocardiographic parameters such as LVEF, LV end diastolic dimension (LVEDD), LV end systolic dimension (LVESD), tricuspid annular plane systolic excursion (TAPSE), mid RV diameter, RA volume, and electrocardiographic parameters such as QRS duration, QTc, and ST‐T changes were measured and recorded before starting chemotherapy according to recommendations by the American Heart Association (AHA). At the end of the study period (6 months), the pre‐ and posttreatment electrocardiographic and echocardiographic findings were collected, compared, and analyzed for all patients.

### 2.1 Statistical Analysis

To summarize the data set, descriptive measures (e.g., averages, standard deviations, and frequency counts) were applied. For statistical testing, a range of methods was employed based on the type and distribution of data. When assessing differences in continuous variables between two independent groups with non‐normal distributions, the Mann–Whitney *U* test was utilized. For comparisons of related samples measured at two time points (pre‐ and post‐intervention), the paired *t*‐test was implemented. For comparisons across more than two independent groups, the Kruskal–Wallis test was selected for analyzing continuous variables. Qualitative (categorical) data were compared using Fisher's exact test due to its suitability for small sample sizes. All statistical computations were conducted in Stata 12 (StataCorp), with statistical significance determined at a two‐tailed alpha level of 0.05.

## 3 Results

A total of 80 cancer patients undergoing chemotherapy regimens of 5‐fluorouracil (20 patients), anthracycline (20 patients), taxotere (20 patients), and herceptin (20 patients) were enrolled. The mean age of the participants was 49.82 ± 8.76 years and 43.75% (35 patients) were male (Table 1).

**TABLE 1:** Demographic and cardiographic characteristics of the patients.

Variable		Frequency/mean ± sd	Percentage
Gender	Male	35	43.75%
	Female	45	56.25%
CVD risk factors	Yes	22	27.50%
	No	58	72.50%
Diastolic Dysfunction	Normal	66	82.5%
	Grade I	14	17.5%
Age		49.82 ± 8.76	
HR		71.95 ± 14.82	
LVESD (mm)		28.63 ± 2.77	
LVEDD (mm)		47.18 ± 2.85	
LVEF (%)		67.08 ± 6.77	
TAPSE (mm)		20.18 ± 3.77	
RA volume (mL/m^2^)		22.76 ± 3.46	
QTc (ms)		419.86 ± 39.04	
QRS (ms)		113.67 ± 12.88	
Mid RV diameter		28.15 ± 3.94	

Table 2 presents the patients' electrocardiographic parameters 6 months after chemotherapy. A significant difference was observed in mean heart rate with respect to chemotherapy drug, with patients receiving anthracycline having a higher mean heart rate (*p*‐value < 0.05). At the end of the study, mean LVESD and LVEDD were significantly elevated in the anthracycline and herceptin group compared to the 5‐fluorouracil and taxotere group (*p*‐value < 0.05). Following treatment, mean LVEF was 64.04% in patients treated with 5‐fluorouracil, 59.25% in those treated with anthracycline, 65.7% in the group treated with taxotere, and 59.75% in patients treated with herceptin, indicating statistical significance (*p*‐value < 0.01). Mean TAPSE at the beginning of the study (20.4 mm) underwent a significant decrease over the course of treatment, reaching 18.01 mm at 6 months (*p*‐value < 0.05). Mean LVEF also decreased from 65.25% at the beginning of the study to 64.05% at 6 months, but did not reach the threshold of statistical significance (*p*‐value > 0.05).

**TABLE 2:** Electrocardiographic and echocardiographic parameters of the participants.

Variable		5‐fluorouracil (*n* = 20)	Anthracycline (*n* = 20)	Taxotere (*n* = 20)	Herceptin (*n* = 20)	*p* value*
HR	Pretreatment	69.1 ± 14.35	77.9 ± 16.01	74.15 ± 13.39	66.65 ± 13.81	0.07
	After 6 months	79.35 ± 14.48	88.45 ± 8.88	84.85 ± 15.68	77.3 ± 14.28	0.013
	*p* value**	0.038	0.019	0.018	0.015	
LVESD (mm)	Pretreatment	28.75 ± 2.95	28.6 ± 2.70	28.95 ± 3.05	28.25 ± 2.51	0.881
	After 6 months	28.1 ± 2.46	29.75 ± 2.89	28.65 ± 1.59	30.6 ± 2.43	0.007
	*p* value	0.410	0.016	0.677	0.014	
LVEDD (mm)	Pretreatment	47.9 ± 2.69	47.75 ± 3.25	46.1 ± 2.46	47 ± 2.79	0.171
	After 6 months	47.6 ± 2.89	50.05 ± 3.06	47.1 ± 2.63	49.5 ± 3.10	0.004
	*p* value	0.749	0.007	0.212	0.013	
LVEF (%)	Pretreatment	65.95 ± 7.50	68.3 ± 7.75	67.4 ± 6.41	66.7 ± 5.48	0.733
	After 6 months	64.05 ± 1.04	59.25 ± 4.39	65.7 ± 0.91	59.75 ± 8.01	0.01
	*p* value	0.09	0.003	0.135	0.002	
TAPSE (mm)	Pretreatment	20.4 ± 3.91	21.2 ± 3.99	19.15 ± 3.51	20 ± 3.64	0.372
	After 6 months	18.01 ± 3.82	17.35 ± 2.75	16.45 ± 2.48	17.01 ± 2.71	0.424
	*p* value	0.026	0.002	0.017	0.014	
RA volume (mL/m^2^)	Pretreatment	22.95 ± 3.56	22.55 ± 3.41	23.05 ± 3.95	22.5 ± 3.10	0.971
	After 6 months	23.25 ± 4.11	23.75 ± 3.59	23.86 ± 4.23	22.84 ± 2.93	0.820
	*p* value	0.814	0.352	0.545	0.761	
QTc (ms)	Pretreatment	415.1 ± 33.36	418.5 ± 40.77	429.01 ± 34.89	416.95 ± 47.14	0.724
	After 6 months	427.5 ± 42.54	428.4 ± 32.61	425.05 ± 32.42	428.78 ± 25.42	0.985
	*p* value	0.362	0.419	0.684	0262	
QRS (ms)	Pretreatment	111.85 ± 14.64	114.65 ± 13.21	113.9 ± 13.01	114.3 ± 11.27	0.886
	After 6 months	115.95 ± 12.26	113.45 ± 10.94	114.75 ± 14.89	115.05 ± 13.47	0.943
	*p* value	0.313	0.781	0.827	0.803	
Mid RV diameter (mm)	Pretreatment	28.79 ± 4.92	27.62 ± 6.12	28.42 ± 4.3	28.96 ± 3.85	0.910
	After 6 months	28.01 ± 3.64	28.68 ± 4.31	30.12 ± 4.68	28.53 ± 4.35	0.803
	*p* value	0.763	0.168	0.106	0.915	

* Kruskal–Wallis test *p* value.

** Paired *t* test *p* value.

**FIGURE 1:**
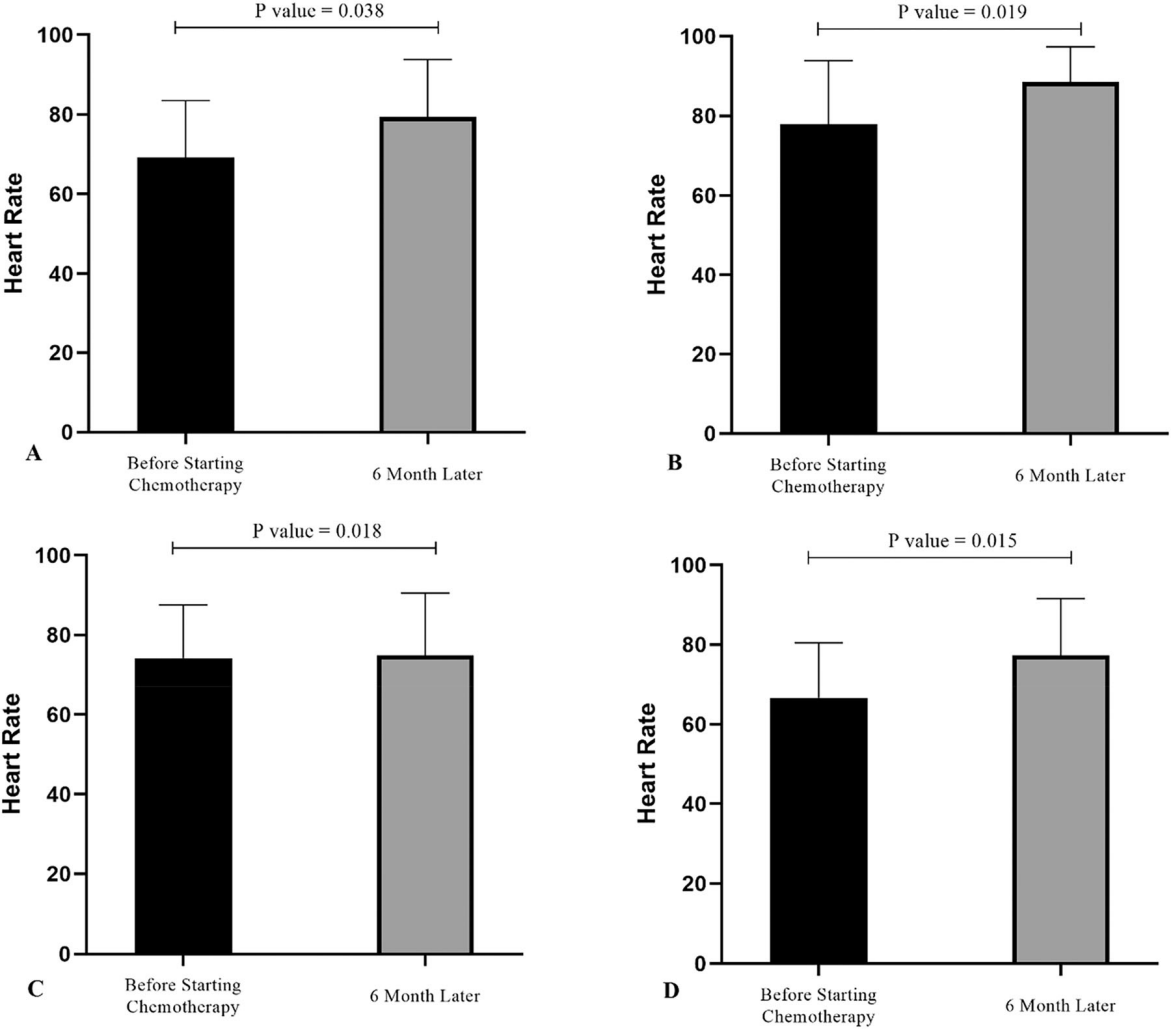
Mean HR in participants before treatment and 6 months after the start of treatment with 5‐fluorouracil (A), anthracycline (B), taxotere (C), or herceptin (D).

Participants treated with anthracycline experienced significantly elevated mean heart rate (HR), LVEDD, and LVESD 6 months after starting treatment (*p*‐value < 0.05). Furthermore, mean LVEF and TAPSE, decreased significantly 6 months after the start of chemotherapy (*p*‐value < 0.01) (Figure 1). In patients treated with taxotere, mean TAPSE was 16.45 mm 6 months after the start of treatment, which was significantly lower than baseline (*p*‐value < 0.05). Mean HR was significantly increased in these patients (*p*‐value < 0.05). Other echocardiographic parameters did not exhibit statistically significant differences following treatment (*p*‐value > 0.05). Patients treated with herceptin had significantly higher mean HR, LVESD, and LVEDD 6 months after the start of treatment (*p*‐value < 0.05). Mean LVEF and TAPSE were significantly lower after 6 months (*p*‐value < 0.01). Table 2 and Figure 1 present the findings for each treatment.

## 4 Discussion

This study examined the impact of chemotherapy on left ventricular function and echocardiographic and electrocardiographic variables in patients undergoing chemotherapy with 5‐fluorouracil, anthracycline, taxotere, or herceptin. The results showed that treatment with anthracycline and herceptin was significantly associated with decreased LVEF and TAPSE, and increased LVESD, LVEDD, and HR. In patients treated with 5‐fluorouracil and taxotere, a significant decrease in TAPSE was observed, while other echocardiographic indices were not impacted significantly.

Several studies explore the effect of chemotherapy drugs on cardiac function and cardiotoxicity [29, 30, 31, 32, 33, 34]. In the study by Nabati et al. [35] on the effects of anthracycline, changes were observed in left ventricular systolic and diastolic function 6 month after the start of treatment and LVEF was significantly lower. On the other hand, LVESD and LVEDD had increased compared to baseline values, aligning with our study, where LVEF decreased while LVESD and LVEDD increased significantly. Appel et al. showed that tissue Doppler and conventional echocardiography could not detect the short‐term effects of low‐dose epirubicin treatment on cardiac function [32]. In our study, EF changed significantly in patients undergoing chemotherapy, indicating the effects of chemotherapy‐induced cardiomyopathy.

In the study by Tan et al. [36] conducted on 29 patients with breast cancer receiving trastuzumab and anthracycline, LVESD and LVEDD increased significantly 24 months after the start of treatment compared to the baseline, which is consistent with our findings. Additionally, strain and strain rate were significantly lower in the 4‐chamber and apical 2‐chamber views compared to pretreatment values. Although the follow‐up period in our study was shorter than Tan et al., the findings indicated a significant increase in LVESD and LVEDD and a decrease in LVEF in individuals treated with anthracycline and herceptin. On the other hand, Kapusta et al. studied children approximately 7 years after chemotherapy and compared them to healthy children, showing that the two‐dimensional echocardiography of these patients did not statistically differ from healthy children. However, their late diastolic wave was lower than healthy individuals in tissue Doppler echocardiography [36]. Using tissue Doppler echocardiography, Amoozgar et al. found echocardiographic changes after treatment with anthracyclines in children, indicating early changes due to anthracyclines that were not observable in two‐dimensional echocardiography [31].

In a study by Gasior et al., it was clearly shown that cardiac toxicity, as assessed by LVEF criteria, occurred in 20 patients (14.3%) during follow‐up, regardless of the treatment method used. Except for the left atrial volume index, diastolic echocardiographic parameters did not show any significant changes in any group after 12 months [30]. In the study by Stoodley et al. [37], Doppler echocardiography, tissue Doppler echocardiography, and two‐dimensional speckle tracking echocardiography revealed changes in diastolic function immediately after treatment with anthracycline, accompanied by a decrease in systolic function. Liang et al. [33] state that LVEF was significantly lower at the end of the study, although the values remained within the normal range. Additionally, the number of patients with abnormal ECG and ST‐T changes increased at the end of the study, consistent with our findings. Other electrocardiographic parameters, such as HR and QTc, also showed significant differences.

Chemotherapy‐induced heart failure following treatment with anthracyclines and herceptin depends on multiple factors, and the reported incidence varies across studies. The incidence of subclinical cardiac manifestations has been reported to range from 0 percent to 57 percent. One of the reasons for this wide variation is the different cumulative doses used in different studies. It has been shown that late‐onset cardiac side effects after treatment with anthracyclines are common and often progressive in cancer patients; for example, cardiac problems after receiving doxorubicin are persistent and progressive [31, 38]. No specific dose has been determined for the onset of cardiac side effects [39], and the reported prevalence of subclinical cardiac side effects differs widely between studies [33].

Myocyte death appears to be the probable mechanism of cardiac injury by chemotherapy drugs such as anthracyclines, and to a lesser extent herceptin, leading to increased serum levels of troponin and NT‐pro BNP. Biomarkers offer a promising means of detecting cardiac damage at a subclinical stage, before the emergence of symptomatic HF. Among these, troponin I stands out due to its high sensitivity and specificity for myocardial injury [40]. Elevated levels of troponin I have been observed not only in patients undergoing conventional chemotherapeutic treatments like trastuzumab and anthracyclines, but also in those receiving more recently introduced therapies, including TKIs [41]. A slight increase in troponin unaccompanied by left ventricular dysfunction does not require discontinuation of the anticancer drug but necessitates careful evaluation, follow‐up, and the addition of cardioprotective drugs. Predicting the cardiotoxicity of anticancer agents, especially those with known cardiotoxicity such as anthracyclines, can be achieved by measuring cardiac biomarkers. However, the timing of the measurement during treatment is not precisely defined. Baseline measurement of biomarkers before initiating anticancer treatment should be considered, which assists with identifying patients at the highest risk of cardiovascular dysfunction, particularly cardiomyopathy and heart failure [38, 39]. The characterization of cardiotoxicity lacks uniformity across the literature, and a globally accepted definition has yet to be established, remaining a topic of ongoing discussion. One definition, proposed by Ky et al., describes cardiotoxicity as either a symptomatic decline in LVEF of ≥ 5% to a value below 55%, or an asymptomatic reduction of ≥ 10% to below the same threshold [16]. More recently, Wenner et al. demonstrated a U‐shaped relationship between LVEF and mortality, with optimal survival occurring at LVEF values between 60% and 65%, suggesting that excessively high ejection fractions may also be associated with increased risk [42].

Typically, cardiac injury is determined by a decrease in LVEF, which includes a decrease of more than 5 percent to less than 55 percent in asymptomatic patients. Determining the baseline EF before each treatment cycle is recommended, especially in high‐risk patients. Patients with a baseline EF of less than 50 percent are at risk of irreversible cardiotoxicity in the course of anthracycline therapy and are advised to utilize other regimens. Anthracyclines are not recommended for patients with an EF of less than 40 percent. Measuring EF and diastolic function before starting potentially cardiotoxic anticancer drugs may help identify individuals at higher risk of cardiovascular disease and should be performed as a baseline measurement [38, 43]. Before initiating treatment with an oral anticancer medication known to CV risks, a thorough assessment of the patient's baseline CV profile is essential. Multiple reviews have underscored the necessity of establishing cardiovascular risk status in this clinical context [44, 45]. Key risk factors include being over 60 years of age, previous coronary artery disease or myocardial infarction, atrial fibrillation, prior HF, smoking, dyslipidemia, hypertension, diabetes mellitus, and obesity. Patients presenting with one or more of these conditions and who are undergoing potentially cardiotoxic oral cancer therapy fall into Stage A heart failure under the American College of Cardiology (ACC)/American Heart Association (AHA) classification, warranting proactive interventions to halt disease progression [46, 47]. As part of the baseline workup, it is generally advisable to obtain an ECG, as well as measure fasting lipid levels and hemoglobin A1C [45]. Patients receiving cardiotoxic anticancer drugs should be considered as having stage A HF (without structural disease and symptoms of HF). Some studies suggest treatment with ACEIs, ARBs, or selective beta‐blockers to mitigate the risk of cardiotoxicity from anticancer drugs in patients with structural heart disease and cardiac risk factors. Several small‐scale investigations have reported favorable outcomes with sacubitril/valsartan in individuals exposed to trastuzumab and/or anthracyclines [48, 49]. GLS, assessed via two‐dimensional speckle‐tracking echocardiography, may offer earlier detection of cardiotoxic changes, appearing nearly 3 months before a measurable drop in LVEF. However, GLS is influenced by image resolution, software‐specific variability, and hemodynamic conditions [19]. A decline in GLS may serve as an early warning signal, prompting the initiation of cardioprotective strategies or even consideration of discontinuing the offending chemotherapeutic agent [4, 38]. In our study, a decrease in LVEF below the normal range occurred in 4 patients 6 months after initiation of chemotherapy, while LVEF decreased but remained in the normal range in other patients.

This study suffers from a number of limitations, including the short follow‐up period, which limited the evaluation of the long‐term effects of the treatments. Additionally, given the high diversity of cancer patients and treatment regimens, it was not possible to specifically investigate cardiotoxicity for each chemotherapy drug or each type of cancer. Finally, a control group was not included in the study, making comparisons with healthy individuals impossible.

## 5 Conclusion

The findings demonstrated that chemotherapy with anthracycline and herceptin was significantly linked to changes in left ventricular function and echocardiographic parameters such as LVESD, LVEDD, and TAPSE. Changes in parameters such as HR and TAPSE were observed in individuals undergoing chemotherapy with 5‐fluorouracil and taxotere, but the changes in other parameters were not significant. Further studies conducting extended follow‐up of the cardiac side effects of chemotherapy drugs are recommended.

## Author Contributions

**Alireza Abdollahi Moghadam:** conceptualization, investigation, funding acquisition, validation, project administration, supervision, methodology. **Seyed Hamed Fazaeli:** investigation, writing – original draft, validation, writing – review and editing, formal analysis, data curation. **Mostafa Kamandi:** conceptualization, funding acquisition, visualization, methodology, project administration. All authors have read and approved the final version of the manuscript Alireza Abdollahi Moghadam had full access to all of the data in this study and takes complete responsibility for the integrity of the data and the accuracy of the data analysis.

## Ethics Statement

This study received approval from the Ethics Committee of MUMS in March 2022 (Code: IR.MUMS.MEDICAL.REC.1401.022).

## Consent

The participants provided written informed consent before enrollemnt.

## Conflicts of Interest

The authors declare no conflicts of interest.

## Transparency Statement

The lead author Alireza Abdollahi Moghadam affirms that this manuscript is an honest, accurate, and transparent account of the study being reported; that no important aspects of the study have been omitted; and that any discrepancies from the study as planned (and, if relevant, registered) have been explained.

## Data Availability

The data that support the findings of this study are available from the corresponding author upon reasonable request.
